# Advances in Clostridial and Related Neurotoxins

**DOI:** 10.3390/ijms232214076

**Published:** 2022-11-15

**Authors:** Sabine Pellett

**Affiliations:** Department of Bacteriology, University of Wisconsin-Madison, 1550 Linden Dr., Madison, WI 53706, USA; sabine.pellett@wisc.edu

The huge advances in genomics and molecular biology in the past two decades have made now an exciting time to study bacterial toxins, in particular, the most potent bacterial toxin known to humankind, botulinum neurotoxins (BoNTs). BoNTs are a large family of protein toxins produced by a diverse and polyphyletic species, *Clostridium botulinum*, and several strains from related clostridial species, including *C. sporogenes*, *C. butyricum*, and *C. baratii* [1,2,3,4]. BoNTs are 150 kDa dichain proteins and are the causative agents of botulism, the potentially lethal human and vertebrate disease [5,6]. As such, these toxins are significant as disease-causing agents and potential bioterrorist agents, but amazingly they also have been adapted as unique, long-lasting, and widely used bio-pharmaceuticals [7,8]. BoNTs are categorized into seven immunologically distinct serotypes, with several subtypes within each serotype [2]. However, in recent years, discoveries of novel BoNTs and potential BoNT homologs in other organisms have challenged this categorization and expanded the family of BoNTs [9,10,11]. While novel BoNTs and homologs are continually being identified by sequencing, only a few have thus far been purified and functionally characterized. Such functional characterization studies of novel and known BoNTs, while challenging due to various factors, including regulatory restrictions, the polyphyletic nature of the species, a relative lack of genetic tools for the organism, and the complex mechanisms of neuron intoxication and pathogenesis, are promising in several ways. Investigations of novel BoNTs to determine their specific biologic characteristics will aid in increasing our understanding of the molecular mechanisms involved in neuronal cell entry, intracellular trafficking, persistence inside the neuronal cell cytosol, and enzymatic cleavage efficiency. Utilizing the continuing developments of genetic methods, which now allow for the construction of recombinant and chimeric BoNTs, site-directed mutagenesis studies allow for the examination of functional effects at the single-amino-acid level. Combined with ongoing structural analyses, such studies are starting to reveal unprecedented new details on molecular mechanisms underlying BoNT potency, duration of action, and distribution in a physiologic system. Together with genomic and bioinformatics analyses, the results from systematic structural and functional studies have the potential to yield an understanding of the evolutionary forces driving the distribution and diversity of this protein toxin family.

This Special Issue aims to highlight some exciting recent advances in expanding our understanding of the molecular mechanisms underlying the pathogenesis and pharmaceutical potential of the large family of BoNTs. The manuscripts published in this Special Issue cover various topics and several toxin sero- and subtypes. Masuyer et al. examined the mechanisms of ganglioside binding by BoNT/E in a structural study, shedding more light on the cell entry strategies of BoNT/E1 versus BoNT/A1 [12]. A faster cell entry rate of BoNT/E versus BoNT/A [13] and distinct neuronal cell selectivity by BoNT/E3 and BoNT/A1 have been described previously [14]. The preferential binding of BoNT/E1 to more complex gangliosides such as disialoganglioside GD1a versus the preferred binding of BoNT/A1 to trisialoganglioside GT1b demonstrated in the study by Masuyer et al. [12] illuminates a potential molecular basis for the observed faster cell entry kinetics by BoNT/E and opens the door for further mutational and functional studies to enhance specific neuronal cell entry rate and the potency of pharmaceutical BoNTs. Interestingly, David et al. describe the discovery of a common small molecule inhibitor to BoNT/A and BoNT/E designed to block the protein receptor binding to synaptic vesicle protein 2C (SV2C) [15]. Despite previous data indicating that BoNT/A preferentially binds the SV2C isoform and BoNT/E preferentially binds the SV2A and B isoforms [16,17,18], aurintricarboxylic acid (ATA) effectively prevented intoxication by both serotypes [15]. In addition to providing a platform for future developments of cross-serotype small molecule BoNT inhibitors as alternatives to antitoxins, this study emphasizes the need to further examine the molecular mechanisms underlying BoNT sero- and subtype-specific cell entry. Since we have not yet resolved the evolutionary forces driving BoNT uptake, maintenance, and diversity in clostridia and related organisms, we may under-appreciate the diversity and functional distinctions of the many members of the BoNT family of protein toxins concerning human pathology and pharmacology. One study in this Special Issue that addresses the horizontal transfer of *bont* genes in clostridia describes the identification of transposable elements associated with BoNT/E5 in *C. butyricum* [19]. Previous studies have indicated the potential horizontal transfer of *bont* genes by plasmid conjugation combined with potential chromosomal plasmid integration events and proposed the descent of *bont* genes from a precursor protein family with adaptation towards developing into toxins producing vertebrate paralysis followed by diversification [1,20,21].

Other studies in this Special Issue focused on the enzymatic light chain domain of BoNTs. They included a basic science study that revealed that one of the VAMP fragments created by BoNT/B and tetanus toxin cleavage inside neuronal cells persists in the neurons for a sufficient time to be detected by specific antibodies raised to the fragment [22]. In previous studies, VAMP cleavage by these toxins has been detected by the disappearance of the intact VAMP, which is difficult to quantify and challenging to detect if partial cleavage occurs. This study by Fabris et al. demonstrates that this BoNT/B and tetanus toxin cleavage-specific anti-VAMP antibody uniquely detects the cleavage fragment but not intact VAMP [22], and with that provides a novel tool enabling much-needed future studies based on the detection of substrate cleavage in cultured cells and in vivo. The study by Gardner et al. utilized the natural divergence of functional and structural characteristics of BoNT/A1 and BoNT/A3 to shed new light on intracellular neuronal localization and trafficking of the light chains of these two toxins [23]. This study provides intriguing data towards our goal of elucidating the molecular mechanisms determining intracellular light chain action and structure–function-specific differences between the various BoNT sero- and subtypes. Finally, Wang et al. applied the viral-based gene delivery of BoNT/A LC combined with peripheral nociceptor-specific expression to examine the selective targeting of peripheral sensory neurons [24]. Interestingly, in cultured peripheral sensory neurons, the expression of the virally-delivered LC expression construct resulted in the efficient downregulation of pain-related genes and pro-inflammatory cytokine expression. While gene therapy was first proposed in 1972 [25] and clinical research began in 1990, gene therapy is still in its infancy. It was not until 2017 that the first gene therapies were FDA-approved to treat genetically inherited blindness and cancer, and many hurdles remain. While most gene-therapy approaches focus on replacing a defective gene, the idea of using this method for long-term pain treatment by expressing BoNT LC selectively in peripheral sensory neurons is intriguing. Continuing basic research on intracellular LC trafficking and persistence is integral to this endeavor.

In summary, it is exciting to see the progress in basic and applied research on the large family of BoNTs, in particular, the increase in basic science research focused on the many naturally occurring variants of BoNTs (Figure 1). As this research progresses, we inch closer to answering the evolutionary significance of these toxins to their host bacteria. Unlocking the door to understanding the underlying molecular and evolutionary mechanisms of the high potency of BoNTs in humans and vertebrate animals is a central goal of BoNT research, which ultimately has the potential to yield novel and improved BoNT-based pharmaceuticals, and lead to improved safety approaches and countermeasures developments to protect humanity from the deleterious effects of these potentially lethal toxins.

## Figures and Tables

**Figure 1:**
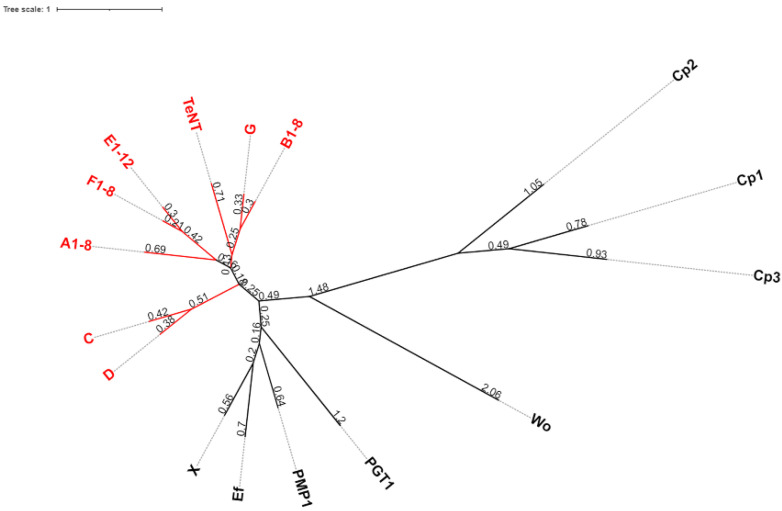
BoNT and homologs. The sequences for BoNT/A1, B1, C, D, E1, F1, G, X, Wo, Ef PMP1, PGT1, Wo, Cp1, Cp2, Cp3, and TeNT were aligned using Clustal Omega (default settings), and a phylogeny tree was created in IQTree [26] using ModelFinder [27] (model of substitution VT + F + G4). For PGT1, the LC and HC sequence were combined into one putative protein sequence. The tree was rendered in iTOL as an unrooted tree. Branch lengths are displayed, and the toxins known to be potent human and vertebrate neurotoxins are displayed in red.
